# Rational Design of Small-Molecule Stabilizers of Spermine Synthase Dimer by Virtual Screening and Free Energy-Based Approach

**DOI:** 10.1371/journal.pone.0110884

**Published:** 2014-10-23

**Authors:** Zhe Zhang, Virginie Martiny, David Lagorce, Yoshihiko Ikeguchi, Emil Alexov, Maria A. Miteva

**Affiliations:** 1 Université Paris Diderot, Sorbonne Paris Cité, Molécules Thérapeutiques In Silico, Inserm UMR-S 973, Paris, France; 2 INSERM, U973, Paris, France; 3 Computational Biophysics and Bioinformatics, Department of Physics and Astronomy, Clemson University, Clemson, South Carolina, United States of America; 4 Faculty of Pharmaceutical Sciences, Josai University, Togane, Japan; Broad Institute of Harvard and MIT, United States of America

## Abstract

Snyder-Robinson Syndrome (SRS) is a rare mental retardation disorder which is caused by the malfunctioning of an enzyme, the spermine synthase (SMS), which functions as a homo-dimer. The malfunctioning of SMS in SRS patients is associated with several identified missense mutations that occur away from the active site. This investigation deals with a particular SRS-causing mutation, the G56S mutation, which was shown computationally and experimentally to destabilize the SMS homo-dimer and thus to abolish SMS enzymatic activity. As a proof-of-concept, we explore the possibility to restore the enzymatic activity of the malfunctioning SMS mutant G56S by stabilizing the dimer through small molecule binding at the mutant homo-dimer interface. For this purpose, we designed an *in silico* protocol that couples virtual screening and a free binding energy-based approach to identify potential small-molecule binders on the destabilized G56S dimer, with the goal to stabilize it and thus to increase SMS G56S mutant activity. The protocol resulted in extensive list of plausible stabilizers, among which we selected and tested 51 compounds experimentally for their capability to increase SMS G56S mutant enzymatic activity. *In silico* analysis of the experimentally identified stabilizers suggested five distinctive chemical scaffolds. This investigation suggests that druggable pockets exist in the vicinity of the mutation sites at protein-protein interfaces which can be used to alter the disease-causing effects by small molecule binding. The identified chemical scaffolds are drug-like and can serve as original starting points for development of lead molecules to further rescue the disease-causing effects of the Snyder-Robinson syndrome for which no efficient treatment exists up to now.

## Introduction

It is well documented that missense mutations can result in various human diseases due to their effects on the structure, function, assemblages, interactions, and other properties of expressed proteins (see for ex. [1]–[6]). Some of these changes are caused by a single mutation in a given protein, other pathologies can be genetically complex, such as the various cardiovascular diseases and cancers with several genes contributing to the disorder [2]–[4]. Frequently, missense mutations causing such disorders affect protein-protein interactions (PPIs) or protein domain interactions [5], [7], [8]. PPIs are essential component of any biological system. As over 370,000 PPIs are predicted to take place within humans [9], the alteration of PPIs is one of the dominant mechanisms by which missense mutations affect the wild type functionality. Recent studies demonstrated [8], [10]–[13] that both disease-causing and harmless missense mutations occurring at the binding epitope do affect protein interactions. However, the magnitude of the effect is difficult to predict because of structural rearrangements and the plasticity of protein-protein interfaces [10], [14]. In a more complex case scenario, one could map the altered PPI into the interactome and consider alternative approaches to restore the interactome, rather than to focus on a particular PPI [15], [16]. During the last decade, initial research has been done to use small organic molecules to act as PPIs inhibitors [17]–[24] or PPIs stabilizers [7], [25]–[29]. However, efficient modulation of PPI by small drug-like molecules is still considered an extremely challenging task, which becomes much more difficult when missense mutations destabilize PPI interactions. In fact, very few examples of direct or indirect stabilizers of mutation altered PPIs have been reported [29]–[32]. For example, in the transthyretin (TTR), several mutations are known to destabilize the TTR tetramer. The TTR tetramer destabilization facilitates amyloid fibril formation causing familial amyloid polyneuropathy. A series of compounds bound to TTR have been found to inhibit the fibril formation via the stabilization of the TTR tetramer [7], [32]. Further, the tumor suppressor p53, a key protein in the cell’s defense against cancer, is deactivated by mutations in 50% of human cancers [33]. Many of the p53 oncogenic mutants are deactivated because their stability is lowered so that the protein denatures very rapidly. Several small molecules stabilizing p53 in a mutation-specific way (e.g. binding to the mutational cavity of p53-Y220C) have been identified by using *in silico* structure-based screening [30] and fragment-based screening [31].

Discovering druggable pockets and identifying small-molecule modulators of challenging protein targets, such as PPI [34] or protein-membrane interactions [35], [36], is not an easy biochemical task. The difficulties can be greatly reduced by utilizing *in silico* approaches, in particular *in silico* screening [37]–[39]. Even some of the hit molecules identified *in silico* do not completely achieve the desired effect, however, they can serve as templates and can be further optimized (e.g. refer to the optimization of survivin dimerization modulators [40]) or can serve as valuable tools for chemical biology goals [37].

**Figure 1:**
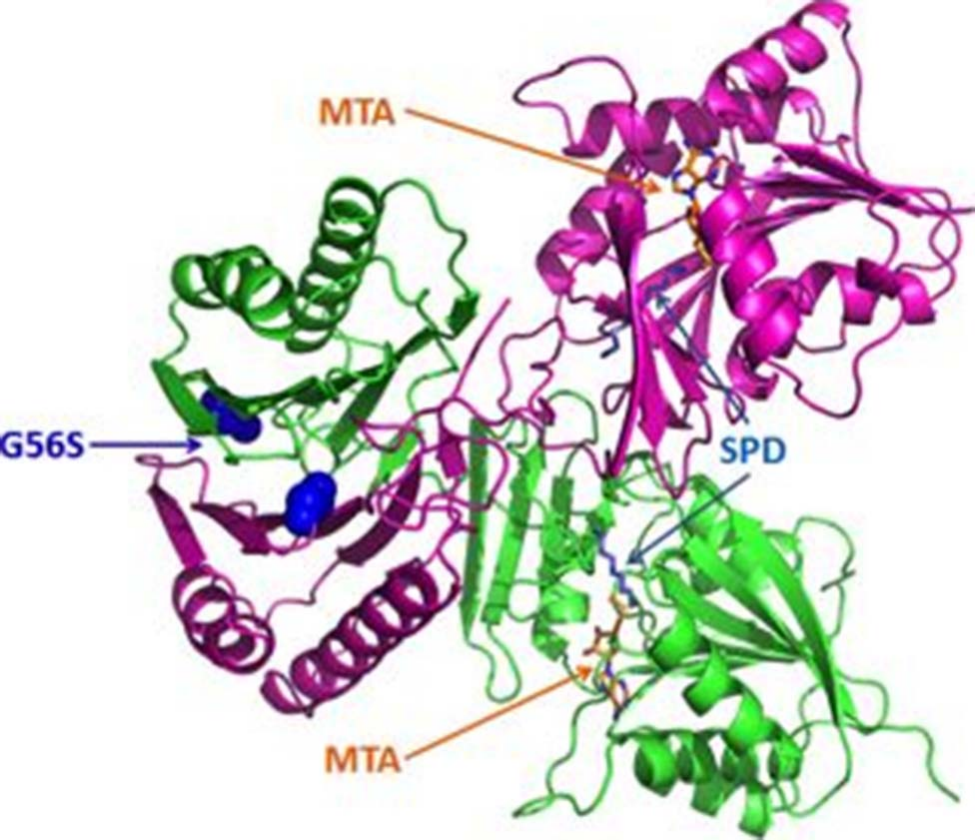
The 3D structure of human SMS (PDB ID: 3C6K). C chain is represented in green and D chain is represented in magenta. The disease-causing mutation G56S is shown in blue spheres; the substrates SPD (sky blue) and MTA (orange) were shown in stick representation.

Here, we report a study focusing on a missense mutation G56S occurring in the vicinity to the homo-dimer interface of the human enzyme spermine synthase (SMS) and causing a rare mental retardation disorder, the Snyder Robinson Syndrome (SRS) [41]–[44]. The SMS forms a homo-dimer with two identical subunits and each subunit has two domains: N-terminal domain (NTD) and C-terminal domain (CTD) (Figure 1). It was shown experimentally that formation of homo-dimer of SMS is crucial for its enzymatic activity [45]. The two NTDs from each subunit contain two large pseudo-symmetric beta sheets forming a dimer interface and harbor the disease-causing missense mutation G56S. It was shown that the G56S mutation greatly reduces SMS activity and leads to severe epilepsy and cognitive impairment [43], along with other currently known missense mutations [43] p.V132G (c.496 T>G) [44], p.I150 T (c.550 T>C), and Y328C [46]. The SMS is involved in the synthesis of polyamines critical for mammalian cell growth and development [47]–[50] by converting spermidine (SPD) into spermine (SPM). The reaction involves an aminopropyl group to be taken from decarboxylated S-adenosylmethionine (dcAdoMet) and transferred to SPD to form SPM and leaving 5′-methylthioadenosine (MTA) as a byproduct. The molecular mechanisms of above mentioned mutations were investigated [11], [12], and specifically we showed, both computationally and experimentally, that the G56S mutation affects the SMS wild type function by decreasing homo-dimer stability. [6], [12]. Since homo-dimerization is known to be crucial for the function of SMS, the disease effect of G56S was attributed to the affected homo-dimer formation [12].

In our previous work we have exploited the possibility to increase the SMS activity by stabilizing the homo-dimer of the SMS mutant G56S through a limited number of small-molecule stabilizers [51]. Here, we extend our previous investigation and designed an original *in silico* protocol-coupling virtual screening and free binding energy-based approach to identify small-molecule candidates capable of stabilizing the G56S homo-dimer. In order to find putative druggable pockets at the mutant dimer interface, we perform molecular dynamic (MD) simulations of the mutant homo-dimer structure combined with a Hierarchical Ascendant Classification (HAC) procedure, which was recently demonstrated to be highly efficient for the identification of a conformational ensemble of pockets [52]. The *in silico* protocol allowed us to successfully prioritize a very small number of candidates for *in vitro* assays starting from more than 2 million chemical compounds. Among the 51 small molecules experimentally tested, 17 showed an increase of the mutant activity, suggesting that their binding stabilizes the SMS G56S homo-dimer. Chemical structure classification allowed to identify five distinct active chemical scaffolds and the structural origins of the stabilization were analyzed by combining molecular docking and MD simulations. The drug-likeness of the identified scaffolds suggests that they may serve as original starting points for the development of optimized lead molecules to further rescue the disease-causing effect of Snyder-Robinson Syndrome.

## Results

### Overall computational procedure

**Figure 2:**
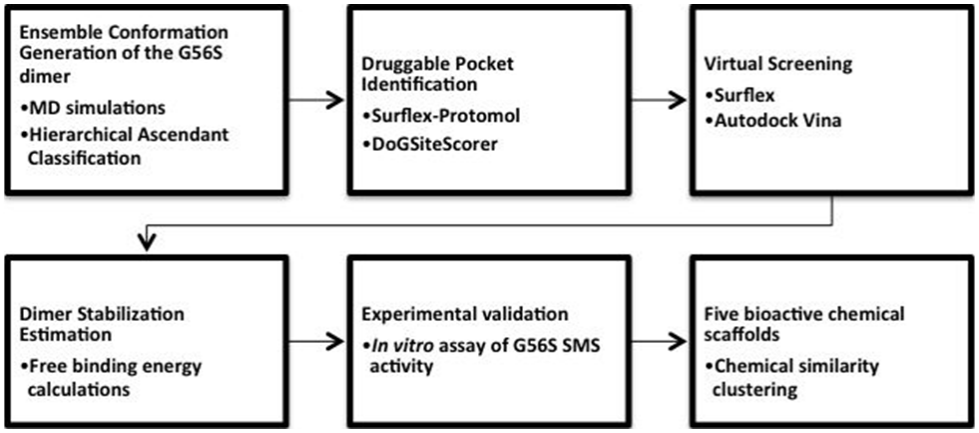
A flowchart of the designed in silico protocol to identify small-molecule stabilizers of the G56S SMS homo-dimer.

The following computational procedure was designed to identify small-molecule stabilizers of the SMS G56S homo-dimer (Figure 2). Details are described in the Methods section. Below we describe the results of each step separately.

### Molecular Dynamics Simulations

We performed molecular dynamic (MD) simulations with 2 ns production step on both the homo-dimer WT and the homo-dimer mutant G56S structures. In order to ensure the reliability of the MD trajectories of the simulated WT/mutant structures, we calculated the root-mean-square deviations (RMSD) of backbone atoms for the entire protein against the average MD structure. The average structure (over 2000 snapshots extracted at each 1ps timestep) was minimized with CHARMM using the same protocol as for the initial minimization. The RMSD of both the WT and the mutant homo-dimers are shown in Figure S1. As expected, the mutant G56S homo-dimer is less stable showing much larger fluctuations than the WT, as observed in our previous studies [11], [12]. After 500 ps of the production step, the RMSD of the WT homo-dimer saturated around 1.5 Å, thus, we took the 1500 snapshots from 500 to 2000 ps at each 1ps timestep for the WT and the mutant for further consideration.

**Figure 3:**
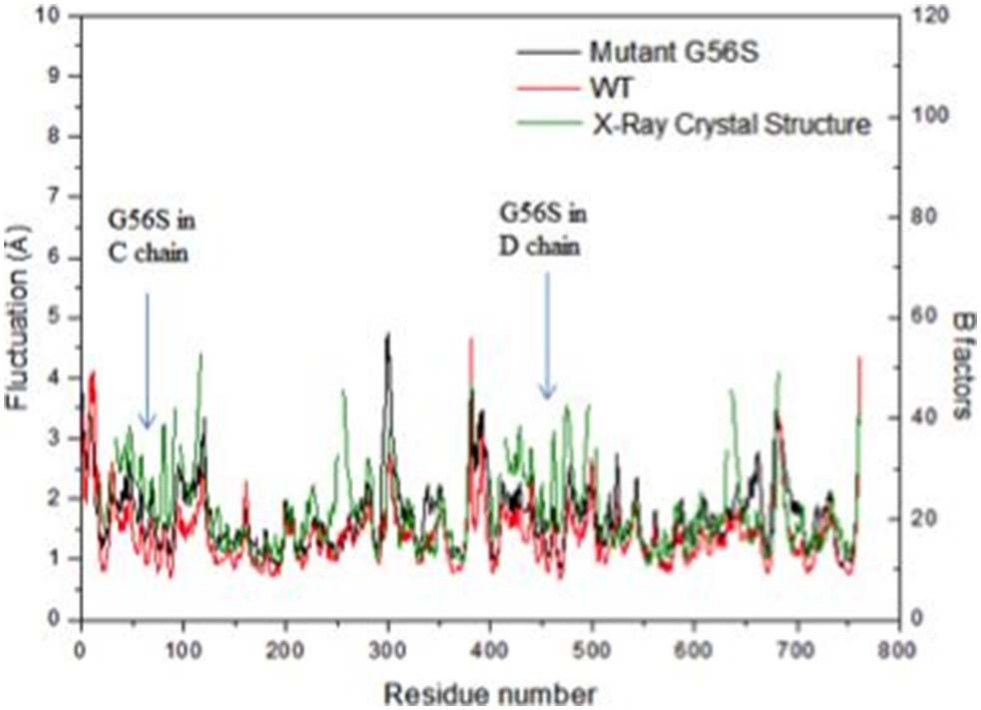
RMSF of simulated WT structure (red) and mutant G56S structure (black); B factors (C^α^ atoms) of the WT X-Ray crystal structure (green). Note that the residue numbers in D chain, which includes 381 amino acids as C chain, were counted from No. 382 to No. 762. The mutation site G56S in both C chain and D chain is pointed to by the blue arrow.

The root-mean-square fluctuations (RMSF) of the C^α^ atoms are shown in Figure 3. For comparison, the B-factors of C^α^ atoms of the SMS WT X-ray crystal structure are also provided. It can be seen that the RMSF of the simulated WT structure are in a good agreement with the B-factors, i.e. the flexible zones observed in the simulated WT structure are similar to those indicated by the B-factors in the X-Ray crystal structure. Since the calculated RMSF closely match the crystallographic B-factors, it can be assumed that the MD simulation trajectories are reliable and can be used in the search for putative druggable pockets for virtual screening. However some differences are noted, e.g. the B-factor of the residues around Lys 250 is higher in the X-ray crystal structure than in the fluctuations of the corresponding residues in the simulated structure. Such differences can be due to the missing residues in the X-Ray crystal structure, which were rebuilt *in silico*. The simulations indicate that the RMSF of the mutant G56S are relatively higher than those of the WT for the entire structure as well as in the region around the mutation site. This observation suggests that the G56S mutation makes SMS homo-dimer more flexible than the WT SMS.

### Identification and Characterization of Druggable Pockets at the Homo-Dimer Interface

**Figure 4:**
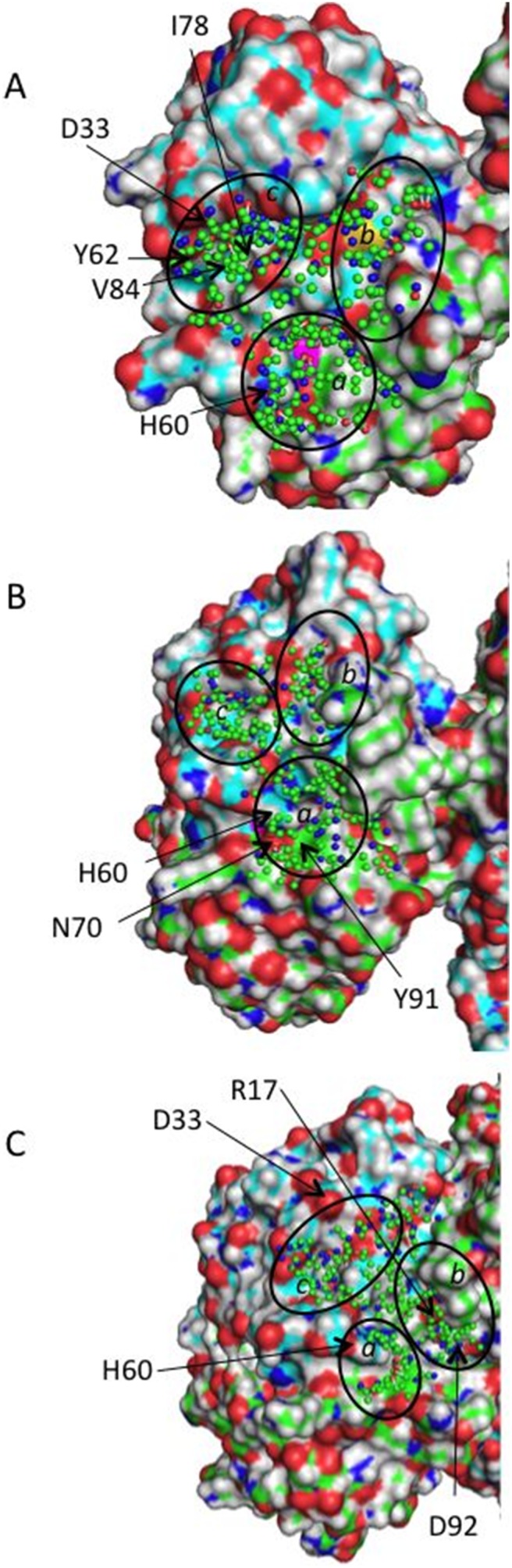
Putative Binding Pockets in the NTD of the Targeted Mutant Dimer Protein Structures. (A) Charmm_mini; (B) Charmm_ave; (C) Charmm_706ps. In the cartoon representations, the green and cyan surfaces represent hydrophobic/aromatic residues for chains C and D, respectively; the red surface represents oxygen atoms; the blue surface represents nitrogen atoms; the magenta surface represents the disease-causing missense mutation; the black circles indicate the subpockets Pa, Pb and Pc.

In order to identify alternative small molecule binding zones at the homo-dimer interface around the mutation site, we analyzed the CHARMM minimized mutant structure, Charmm_mini, and the minimized average mutant structure of the entire MD production trajectory, Charmm_ave, using protomol probes generated by Surflex. Such an analysis would allow the discovery of transient druggable pockets at the dimer interface in different conformations that would permit the performing of virtual screening into alternative cavities and to discover small-molecule binders with different chemistry. The analysis of the two N-terminal domains (NTD) of both chains in the homo-dimer for Charmm_mini (Figure 4A) suggests three cavity candidates (termed subpockets), Pa, Pb, and Pc, which are close to the mutation site. Subpocket Pa is mostly formed by residues from the C chain and it is the largest and most hydrophobic one among the three cavities. Subpocket Pb goes across the dimer interface and is linked through small channels to both subpockets Pa and Pc. Subpocket Pc is located at the D chain and contains several hydrophobic residues (A32, Y62, I78, V84) and two negatively charged side chains D33 and E35. The minimized average MD structure Charmm_ave also suggests three subpockets Pa, Pb and Pc (Figure 4B). Subpockets Pa shows different geometries and polarities in Charmm_ave and in Charmm_mini. In Charmm_ave, subpocket Pa goes along with the dimer interface towards the subunit C. The mutation site G56S is located within the deep cavity of this subpocket. Several polar hot-spot residues are located in subpocket Pa (H60 from the chain D and N70 form chain C) creating a strong polar environment. The aromatic Y91 (C chain) provides a possibility for aromatic/hydrophobic contacts with an incoming ligand. Subpocket Pc includes the same charged residues as subpocket Pc in Charmm_mini. In both structures subpocket Pc is far from the dimer interface. Considering the different polarity and shape of the subpockets Pa and Pb in Charmm_ave and in Charmm_mini we retained these zones as putative binding sites that could accommodate diverse ligands as homo-dimer stabilizers.

In order to find different conformations of the identified putative binding sites, we employed Hierarchical Ascendant Classification (HAC) based on the matrix of RMSD for all atoms of the putative binding pockets of the 1500 MD extracted snapshots of the mutant homo-dimer. This procedure resulted in 8 homo-dimer conformations with diverse binding pockets. In order to select the best druggable structure we performed druggability analysis using the DoGSiteScorer webserver for the obtained 8 centroid homo-dimer conformations (see Table S1). Among the 3 best structures (706ps, 790ps and 1353ps) having pockets close to the mutation site G56S with druggability score >0.80, we retained the conformation 706ps, Charmm_706ps, having the druggable cavity with the biggest volume close to G56S.

In order to analyze the population density of the conformation 706ps we calculated RMSD between the 1500 conformations used for the HAC analysis and Charmm_706ps (Fig. S2). Only 10 structures were found to be very similar to Charmm_706ps with RMSD within 1.5 Å. Such result can be expected because the HAC clustering is done only over the putative binding site residues in order to find diverse binding site conformations to dock ligands into. Thus, the obtained centroid structures may not be really considered as representative for the conformational population of the entire homo-dimer mutant structure.

**Figure 5:**
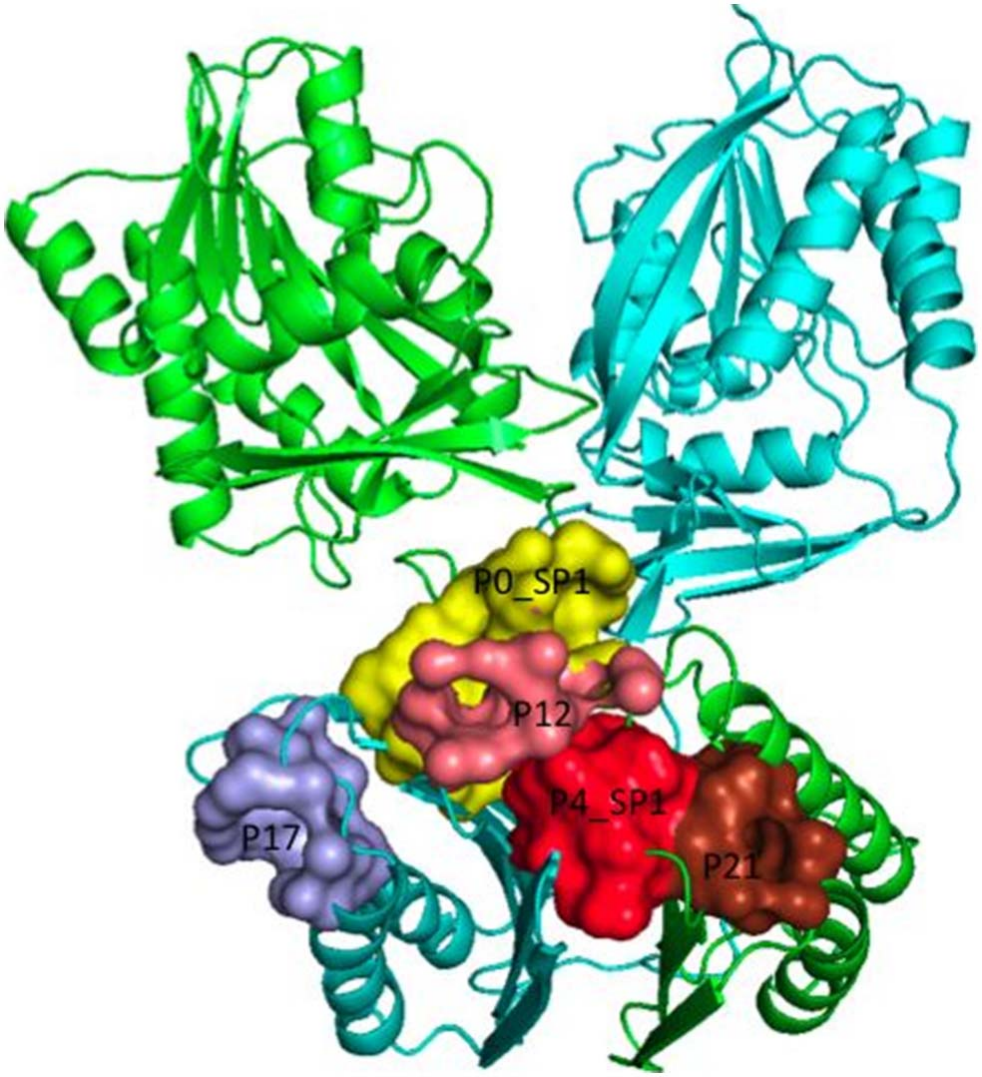
Druggable pockets (P) and subpockets (SP) close to the targeted dimer interface of Charmm_706ps identified by DoGSiteScorer.

Table 1 shows the druggability scores and calculated descriptors for the best druggable pockets at the entire surface of Charmm_706ps identified by DoGSiteScorer. The pockets P0 and P4 situated around the homo-dimer interface (see Figure 5) show high druggability scores of 0.81 and 0.84, respectively. Then, we used the Surflex protomol tool to analyse the druggability of Charmm_706ps. We obtained three subpockets Pa, Pb and Pc for Charmm_706ps (shown in Figure 4C). The subpockets Pa and Pb have a surface covering the dimer interface larger than in Charmm_ave, suggesting that small molecules bound in these subpockets may result in stabilization of the homo-dimer mutant. Table 2 shows all subpockets of Charmm_mini, Charmm_ave and Charmm_706ps closely placed to the targeted homo-dimer interface. In fact, the subpocket P4_SP1 (subpocket 1 of pocket P4) and pocket P21 of Charmm_706ps correspond to the subpocket Pa shown in Figure 4C. The subpocket P0_SP1 and pocket P12 of Charmm_706ps correspond to the subpocket Pb shown in Figure 4C. As seen from Table 2, the highest druggability score is obtained for the area Pc of Charmm_ave, yet it is located too far from the dimer interface. Among the three mutant protein conformations, the best druggability score for a pocket close to the dimer interface corresponds to the druggable area Pb of Charmm_706ps. Finally we retained the druggable areas Pb and Pb of the structures Charmm_706ps, Charmm_mini and Charmm_ave, which are closely placed to the homo-dimer interface and show different geometries and polarities for virtual screening experiments.

**Table 1:** Druggable pockets (P) identified by DoGSiteScorer at the entire surface of Charmm_706ps.

Pocket	Volume [Å^3^]	Surface [Å^2^]	Solvent accessible lipophilicsurface [Å^2^]	Drugability Score
**P0**	**1490.59**	**1875.12**	**1296.21**	**0.81**
P1	835.29	1097.69	777.25	0.84
P2	767.53	865.98	523.04	0.84
P3	702.28	812.86	578.89	0.84
**P4**	**536.92**	**458.24**	**284.69**	**0.84**
P5	504.30	677.41	364.36	0.79
P6	464.84	605.06	410.05	0.86
P7	411.02	889.07	556.32	0.72
P8	348.84	482.93	347.50	0.69

**Table 2:** Druggable pockets (P) and subpockets (SP) identified by DoGSiteScorer close to the targeted dimer interface of Charmm_mini, Charmm_ave and Charmm_706ps.

Pocket	Volume	Surface	Solvent accessiblelipophilic surface	Drugability
	[Å^3^]	[Å^2^]	[Å^2^]	Score
***Charmm_706ps***				
***Pa:*** ** P4_SP1**	131.62	394.80	87.07	0.34
** C chain:** R8–R11, S62–F64, Q80–Y82				
** D chain:** W57, R77, Y79, L85				
** P21**	140.82	191.20	96.04	0.31
** C chain:** F36, Q39, M41, N59, S63,F64, A65, L79, Q80, S81, E91, I92, I95				
***Pb:*** ** P0_SP1**	478.78	846.01	509.65	0.53
** C chain**: I120–Y129				
** D chain:** D25, F26, M27, L83–L85,R122–K124				
** P12**	217.08	454.07	335.51	0.51
** C chain:** L5–G9, D83, V121,G123–A125				
** D chain:** M27, H81, L83				
***Pc:*** ** P17**	146.26	326.75	209.65	0.29
** D chain:** L28, A30, K31, D33, T36,I37, E114–Q117, S119, T120				
***Charmm_ave***				
***Pa, Pb:*** ** P11**	221.20	270.06	156.81	0.49
***Pc:*** ** P8**	257.00	516.80	331.28	0.58
**Charmm_mini**				
***Pa*** **: P18**	143.59	172.91	103.05	0.30
***Pb*** **: P2_SP0**	328.41	498.23	344.01	0.40
***Pc*** **: P9_SP1**	102.88	173.25	100.11	0.37
** P9_SP2**	87.17	214.45	98.75	0.06

### Virtual Screening and Free Binding Energy Calculations

In order to identify putative small-molecule stabilizers of the G56S mutant homo-dimer we performed structure-based virtual screening of a compound collection of 273,226 diverse drug-like molecules prepared from more than 2 million chemical compounds. The molecules were docked into the identified putative binding pockets Pa and Pb of Charmm_706ps, Charmm_mini, and Charmm_ave structures using Surflex and AutoDock Vina. The protein conformations were maintained as rigid during the docking computations. For each protein conformation, an independent consensus scoring was performed on the top 2000 compounds ranked by Surflex and AutoDock Vina. 214 common top-ranked compounds were found in all. We found 63 common molecules with the best scores ranging from 6.8 to 8.75 for Surflex and from −7.0 to −8.3 for Vina when docking into Charmm_mini. For Charmm_ave, we found 71 common molecules with the best scores ranging from 7.4 to 9.0 and from −7.7 to −8.6 for Surflex and Vina, respectively. For Charmm_706ps, we found 80 common molecules with the best scores ranging from 7.1 to 8.6 and from −7.3 to −8.3 for Surflex and Vina, respectively. After an interactive visual analysis (focused on shape, hydrophobicity, and polar complementarity) we selected 95 molecules and 2 different binding modes for each ligand that are the most likely to occur as predicted by the docking into Charmm_mini, Charmm_ave, and Charmm_706ps.

To probe the stabilizing effect of the selected 95 ligand candidates, we decided to compute the binding affinity between the homo-dimer protein and the small molecules bound at the homo-dimer interface. Two different protocols based on MD simulations were employed to compute the binding affinities for the G56S dimer-ligand complex, ΔΔG_bind_ and ΔΔG_bind-relaxed_ (see Methods for details). We ranked the 95 ligand candidates by ΔΔG_bind_ and ΔΔG_bind-relaxed_ and the first 51 best ranked ligands with binding affinity ΔΔG_bind_ or ΔΔG_bind-relaxed_ better than −20 kcal/mol were selected for experimental validation.

### *In Vitro* Characterization of the Putative G56S SMS Stabilizers

**Figure 6:**
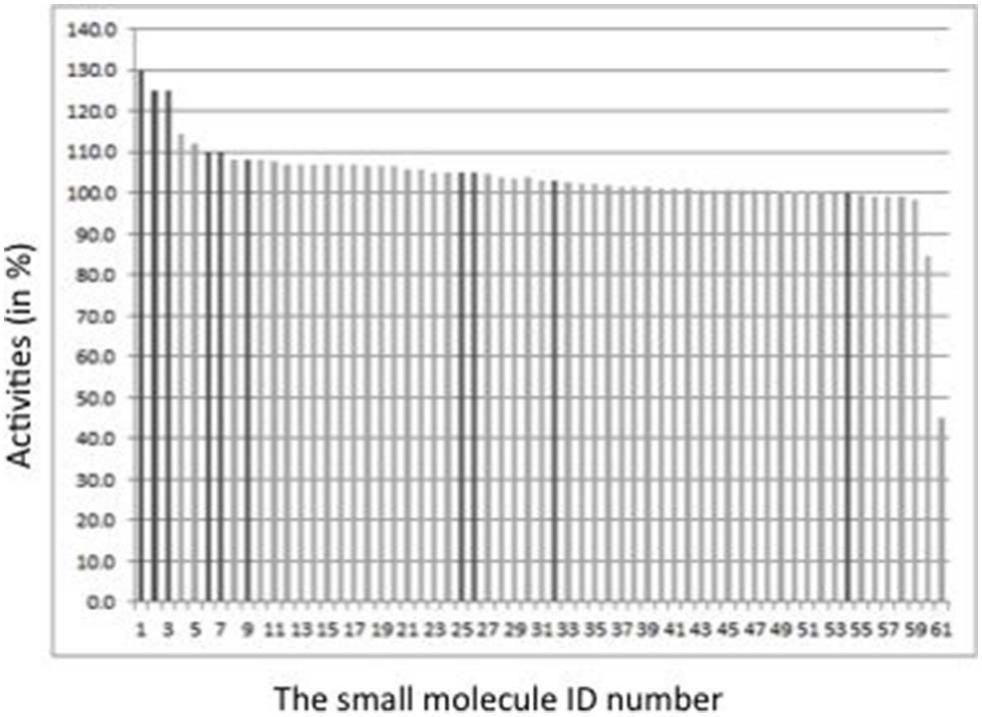
Activity of small molecules experimentally tested. The vertical axis of the graph shows activity normalized to 100% for the G56S SMS mutant without the binding of small molecules. The horizontal axis indicates the small molecule ID number. The newly tested here 51 molecules are shown in grey and the previously tested 10 molecules [51] are shown in black.

The selected 51 compounds were purchased and tested experimentally. The goal of the *in vitro* experiments was to test the putative stabilizers for their ability to increase the G56S SMS activity via the homo-dimer stabilization. The measured activity of G56S SMS in presence of small molecules is shown in Figure 6. The activity in the presence of the previously tested 10 small molecules [51] is also given in Figure 6. It is seen that 31 molecules slightly increase the SMS mutant activity and 7 of them increase the activity of the G56S SMS by more than 10%. Unexpectedly, we discovered two molecules that decrease the mutant activity by 15% and 56%, respectively. One may speculate that these molecules affect the dimer formation or stability since they do not to contain scaffolds known to inhibit the SMS active site and are neither reactive nor frequent hitters that might result in false positive hits (we checked by our software FAFDrugs2, see in Methods for details).

### Binding Affinity Analysis of Mutant Homo-dimer Stabilizers

An analysis of the predicted binding affinities for all 61 molecules is presented below. The calculated scores of Surflex and Vina do not show a correlation between the experimentally found activities and the calculated scores, e.g. the scores do not distinguish the good (with activity >110%) from the bad (activity <110%) binders (results not shown). These results can be expected by taking into account that the reliable prediction of binding affinities still remains an important challenge in structure-based virtual screening methodology [53]–[55]. Current scoring functions are widely recognized to lack precision in accounting for the solvation and entropic contribution to ligand binding. Binding free energy calculations can thus help to prioritize potential binders. Although we did not find a strong correlation between the experimental activities and the computed ΔΔG_bind_ or ΔΔG_bind-relaxed_ energies, we should note that the for the best activators (activity≥110%), better binding energies are computed using the ΔΔG_bind-relaxed_ than using the ΔΔG_bind_ approach (results shown in Figure S3). These results confirm the importance of considering the protein flexibility before and after ligand binding in order to improve the affinity prediction [56]. The binding free energy calculations allowed for the reduction of twice the number of compounds selected after docking-scoring (from 95 to only 51) for the experimental assays.

**Figure 7:**
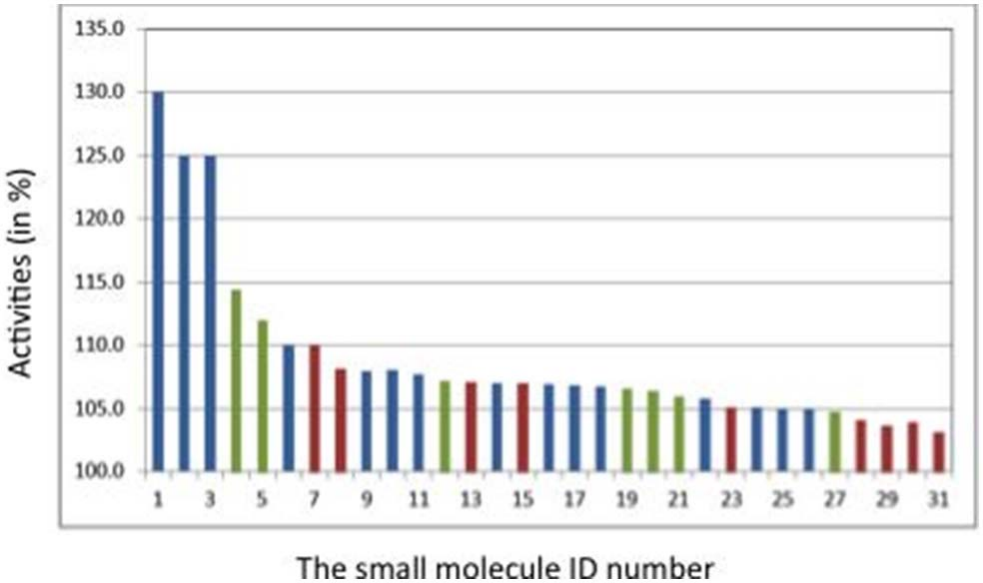
Activities (in %) of the 31 hit molecules identified by docking into the three receptor conformations: Charmm_mini (in blue bars), Charmm_ave (in red bars), Charmm_706ps (in green bars). The two newly discovered here most potent compounds (no 4 and 5) representing 2 new scaffolds are found by docking into the MD snapshot Charmm_706ps.

Figure 7 shows the SMS protein conformations (Charmm_mini, Charm_ave and Charmm_706ps) which were used to identify each experimentally validated hit. The previously identified active molecules (no 1, 2, 3) have been discovered by using docking into the minimized SMS G56S structure (Charmm_mini). Most of the molecules identified by docking into Charmm_ave show slight activity suggesting that the average MD structure (Charmm_ave) is not the most suitable for putative binder identification. Interestingly, the two newly discovered here most potent compounds (no 4 and 5 with activities 114.4 and 112%, respectively) which contain 2 new scaffolds (see next paragraph) were found by docking into the snapshot Charmm_706ps which shows the best putative druggable pocket. This indicates that our procedure of classifying diverse putative binding sites of G56S SMS homo-dimer using MD simulations is useful for identifying druggable binding pockets. In fact, different scaffolds were discovered thanks to docking into diverse binding site conformations.

### Structural analysis of the bioactive molecules

**Figure 8:**
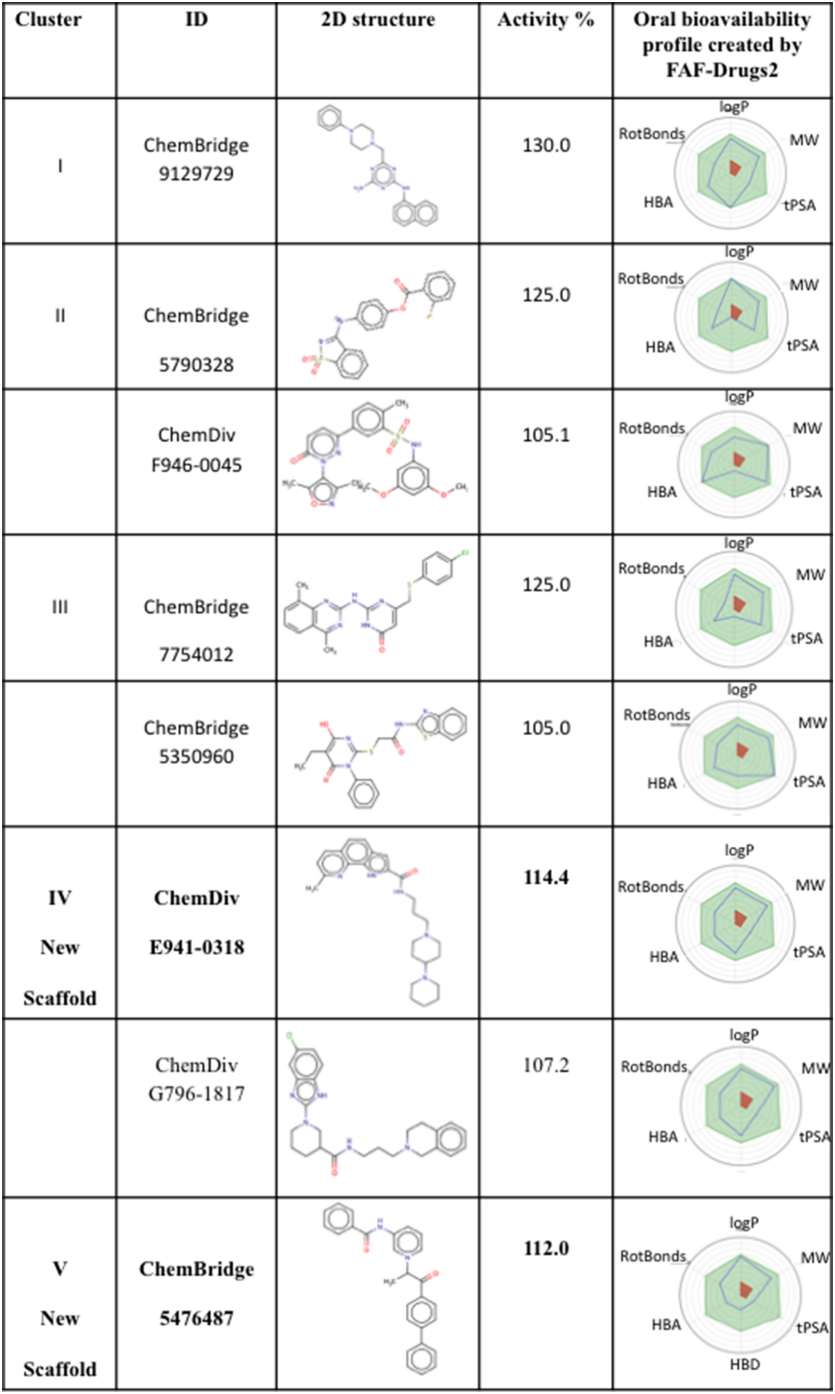
Chemical series of the identified bioactive compounds. Radar plots represent the computed oral bioavailability profile (compound blue line should fall within the optimal green area, white and red ones being extreme zones generally indicating low oral bioavailability). The computations involved: logP, molecular weight (MW), topological polar surface area (tPSA), rotatable bond (RotBonds), H-bonds acceptors and donors (HBA, HBD).

In order to identify a diverse chemical series we performed a chemical similarity search and clustering on 26 bioactive molecules showing increased activity ≥105%. The obtained most active chemical series, which contained at least one molecule with activity >110%, are shown in Figure 8. The first 3 scaffolds (clusters I, II, and III) are represented by the ChemBridge molecules ID: 9129729, 5790328 and 7754012 and have been identified during a previous virtual screening performed on the Charmm_mini structure [51]. Two new scaffolds represented by the molecules ChemDiv ID: E941-0318 and the ChemBridge ID: 5476487 (clusters IV and V) are identified here by docking into the snapshot structure Charmm_706ps. The physicochemical profiles of all compounds seen in Figure 8 satisfy the physicochemical criteria for oral bioavailability. Furthermore, the molecules shown in Figure 8 do not contain reactive groups, frequent hitters or PAINS (Pan Assay Interference Compounds) (verified using FAF-Drugs2) suggesting that these molecules might be specific binders for our target.

**Figure 9:**
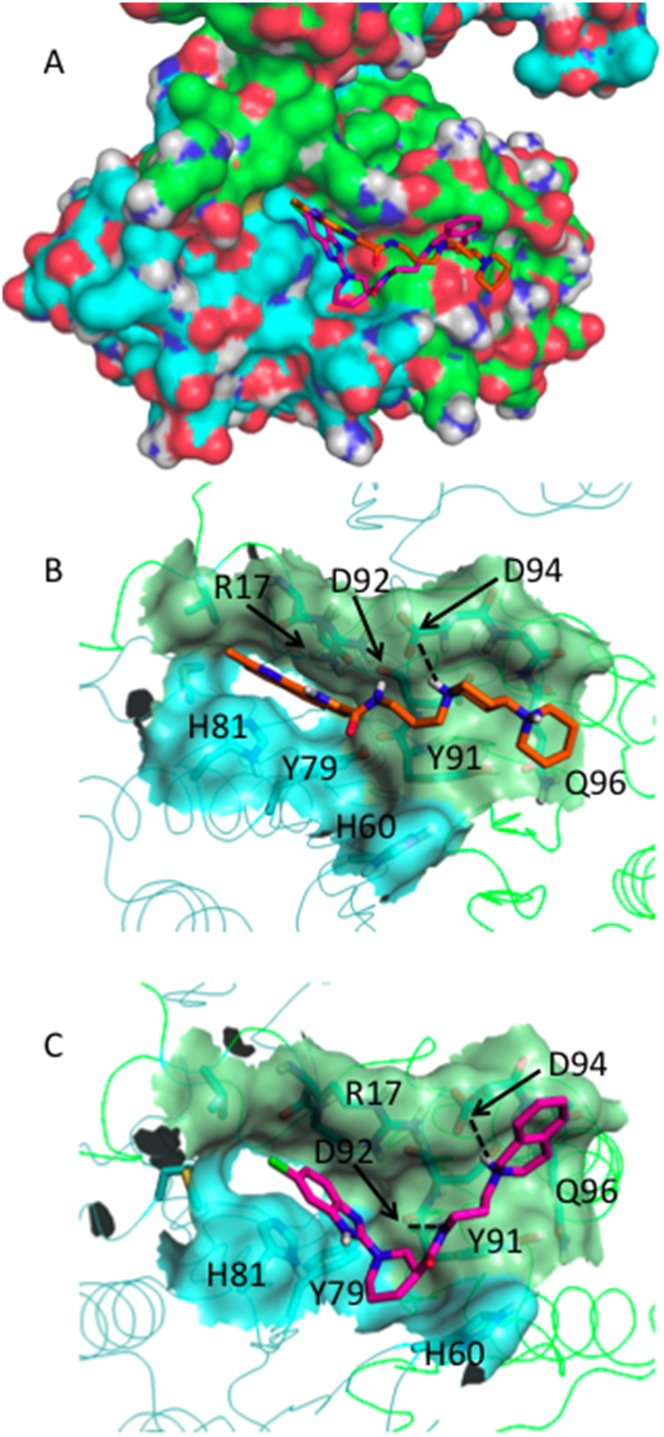
Lowest docking energy conformations of Cluster IV bioactive compounds docked with AutoDock into the area Pb of Charmm_706ps taken after the MD simulation of the complex Charmm_706ps - E941-0318. The C chain in shown in green, the D chain is shown in cyan. (A) docked E941-0318 and G796-1817 superposed into the Connolly surface of the dimer G56S SMS; (B) docked E941-0318; (c) docked G796-1817.

In order to propose a possible mechanism of action for the newly discovered scaffold Cluster IV, we re-docked the two ChemDiv molecules E941-0318 and G796-1817 into the Charmm_706ps putative binding pockets Pa and Pb. For these docking experiments, we took the last protein structure of the G56S dimer of the MD simulation of the complex Charmm_706ps-E941-0318. The lowest docking energy poses suggesting similar orientations for E941-0318 and G796-1817 were obtained in the putative binding area Pb (Figure 9) with docking energies of −7.73 and −7.59 kcal/mol, respectively. In the putative binding area Pa (Figure S4) the lowest docking energies were of −7.95 and −6.94 kcal/mol for E941-0318 and G796-1817, respectively.

The docking experiments shown in Figure 9 suggest that the end of the propyl side chain, namely the first cycled amine in E941-0318 and the cycled amine in G796-1817 point toward the carboxylic group of D94. pKa calculations performed with MarvinSketch software (ChemAxon 2010) predicted that this cycled amine is protonated for both E941-0318 and G796-1817. Thus, the charged cycled amine of E941-0318 and G796-1817 forms a salt bridge with the carboxylic group of D94. In addition the amide NH of E941-0318 and G796-1817 forms a hydrogen bond with the carbonyl oxygen of D92. The aromatic cycles of both molecules are anchored in a deep cavity formed by R17 and H81, Y79. The docked pose of G796-1817 suggests that its Cl atom is in contact with H81. Although it is not exactly situated between the two nitrogen atoms ND1 and NE2, a halogen bond may be expected because of the short distance between ND1 and NE2 and the Cl atom. The present data suggests that the small molecules E941-0318 and G796-1817 fit into the Pb binding pocket, protrude at the molecular surface, and could indeed stabilize the protein-protein interactions at the dimer interface and could thus increase the G56S SMS activity as supported by the experimental validation. Interestingly, H81, Y79, and Y91 have also been proposed to be involved in ligand binding for the previously identified bioactive molecules Chembridge 9129729, 5790328 and 7754012 by docking into the Charmm_mini conformation. Therefore, the previous and the obtained here docking results strongly support the potential binding areas Pa and Pb can be successfully targeted in order to develop small-molecule stabilizers at the G56S SMS dimer interface.

The stabilization effect due to ligand binding is also supported by the performed MD simulations of the mutant G56S bound to identified actives. The RMSF of the mutant G56S, the mutant bound to the newly identified compound E941-0318, and the mutant bound to the previously discovered compound 9129729 (shown in Fig. S5) suggest that ligand binding indeed stabilizes the homo-dimer at the targeted interface. The zone of the residues Y91-T120 of C chain including Y91, D92, D94 and Q96 and that around the key residue H60 of D chain, all expected to be key for the interaction, show reduced fluctuations upon ligand binding.

## Discussion

This work focuses on the missense mutation G56S causing malfunctioning of the enzyme spermine synthase and resulting in the Snyder-Robinson Syndrome. Our previous computational and experimental studies [11], [12] showed that G56S destabilizes the SMS homo-dimer without affecting the active site of the enzyme. Homo-dimer formation is crucial for the normal enzymatic activity of SMS [45], and thus our goal was to mitigate the effect of G56S in order to rescue the dimer affinity. Moreover, G56 is situated in a solvent accessible zone and far from the active site, thus binding a small molecule around the mutation site would have a low risk of affecting the active site. This provides an opportunity to develop an approach aimed at restoring the enzymatic activity of G56S SMS by stabilizing the G56S mutant homo-dimer. *In vivo*, where the SMS molecules are surrounded by many other molecules in the cell, the small molecule binding pockets may not be always exposed to the solvent due to transient interactions with other molecules in the cell. However, these transient interactions are short-lived, since SMS is known not to have interacting partners and therefore the small molecules are expected to be able to reach the pockets without much obstruction. Much more crucial is the question of unwanted binding of the small molecules to other off-targets different from SMS, which often occurs in the cell.

Stabilizers of PPIs can act by variety of potentially complex mechanisms. For instance, small-molecule binding can be used to tackle or stabilize transitory complexes [28] or by targeting allosteric pockets it can also be useful for stabilizing proteins or PPIs in some cases [57]. Thus, the first challenge that should be addressed when targeting PPIs by small drug-like molecules is to identify potentially druggable pockets [34]. It has been recently shown that protein interaction sites are more predisposed to surface pocket formation than the rest of the protein surface [58]. This suggests that the more direct way would be to directly target the PPI interface or domain-domain interface. Some example cases are the small-molecule stabilizers of the TTR tetramer [32] or the dimer of human survivin [26], [40]. With this, our strategy was to identify druggable pockets in different conformations at the homo-dimer interface of G56S SMS. In our previous work [51], we have targeted the homo-dimer interface close to the mutation site in a conformation obtained after a molecular mechanics minimization (Charmm_mini). This resulted in the identification of the molecules, ChemBridge 9129729, 5790328, and 7754012, as stabilizers of the G56S SMS homo-dimer. In order to identify new scaffold molecules, we explored conformational changes that can occur at the mutant homo-dimer interface through MD which would permit us to find transitory pockets. Putative druggable pockets at different modeled conformations were identified in the vicinity of the mutation site G56S based on the consensus results for druggability obtained by two different approaches, Surflex-protomol and DoGSiteScorer. The best performing identified pocket was at the MD snapshot Charmm_706ps, which allowed the identification of two new stabilizing scaffolds: the molecules ChemDiv E941-0318 and ChemBridge 5476487. The 5 distinct scaffolds identified in this work and in our previous one suggest that druggable pockets exist close to mutation sites at PPIs interfaces, which can be successfully targeted via small-molecule binding.

As a proof-of-concept, we combined structure-based virtual screening and conformational and binding energy analysis via MD simulations to identify small molecules that increase the activity of G56S SMS through the mutant homo-dimer stabilization. The successfully identified molecules that increase the G56S SMS activity suggest that the employed computational strategy to explicitly incorporate protein-ligand dynamics into the final selection of compound candidates has successfully allowed for the prioritization of the putative homo-dimer interface binders. Starting from ∼2 million *in silico*-analyzed compounds, we tested 51 compounds experimentally, among them, 23 compounds were found to slightly increase the G56S SMS activity. Binding free energy calculations after the MD simulations helped to identify potential binders. In fact, the best free binding energies for the best active molecules (increasing the G56S SMS activity by ≥110%) were obtained when conformational flexibility of the protein–ligand complex and of the protein alone were taken into consideration. There results confirm the gain in virtual screening accuracy when protein flexibility is incorporated compared to using scoring functions relying on static conformations of protein-ligand complexes, as previously observed [59]–[63]. This observation must be much more valuable when missense mutations are present and destabilize proteins or PPIs or domain-domain interactions. In general, missense mutations increase the conformational space of proteins or their complexes and targeting druggable pockets in different conformations can be helpful to identify different scaffold molecules binding at the PPI or dimer interfaces, as it was demonstrated in this study.

In conclusion, the identified five different scaffolds represent drug-like molecules without potential reactive or PAINS groups, which provides a basis for further optimization of these molecules in order to develop lead therapeutics for Snyder-Robinson syndrome for which no efficient treatment exists until now. Our results confirm that the protein conformational analysis and structure-based virtual screening is a promising approach to target PPI interfaces with present mutations by drug-like molecules to modulate PPI for drug discovery and chemical biology projects.

## Materials and Methods

### I. *In silico* modeling

#### Protein Structure

The X-Ray crystal 3D structure of wild type (WT) human SMS in complex with spermidine (SPD) and 5-methylthioadenosine (MTA) (PDB ID: 3C6K) (Figure 1) was downloaded from the Protein Data Bank (http://www.rcsb.org) [64]. The crystallographic structure is made of four chains (chains A, B, C, and D) resulting in two homo-dimers in the asymmetrical unit cell. As pointed out in our previous work [11], [12], the homo-dimer formed by the A chain and B chain is not suitable for MD simulations because of significant van der Waals clashes. Due to this, in this work, we used the dimer formed by the C and D chains. The missing atoms and residues were rebuilt by “profix”, a module in Jackal package (http://wiki.c2b2.columbia.edu/honiglab_public/index.php/Software:Jackal_General_Description). The mutant G56S was created by the module SCAP [65] in the Jackal package. Figure 1 shows the WT 3D structure and the mutation site G56. In this paper, we kept the original residue number 56 according to previously published papers [43], [45] for the mutation site G56 (G71 in FASTA sequence) while the other residue numbers mentioned in this paper correspond to the protein sequence in FASTA file. The protonation states of the titratable groups were calculated with Multi Conformation Continuum Electrostatics (MCCE, version 2.4) [66]–[68]. The dielectric constant for MCCE was 8.0. The results of pKa calculation suggested that several His (Table S2) are neutral and the hydrogen atoms of these His (H_δ_ or H_ε_) were placed according to the obtained pKa values.

#### Molecular Dynamics Simulations

MD simulations were performed for the WT and the mutant homo-dimer structures using CHARMM program (Chemistry at HARvard Macromolecular Mechanics, version c35b1) [69]. The substrates (SPD and MTA) in SMS complex were removed for the simulations since they are situated at the C-terminal domain (CTD) far from the mutation site G56S located at the N-terminal domain. The solvation was taken into account by the Generalized Born implicit solvent function FACTS [70]. The WT and the mutant homo-dimer structures were initially minimized using 500 steps of a steepest descent algorithm followed by 500 steps of a conjugate gradient algorithm. Distances between heavy atoms and hydrogen atoms were constrained by the SHAKE algorithm allowing for a time step of 2 fs. The system was heated during 100 ps to reach 300 K and then equilibrated during 200 ps with a temperature window of 300±10 K. The production time was 2 ns for each MD simulation run. Based on the MD analysis, we found that the long NTD tail of 9 amino acids of the mutant dimer is extremely flexible and might cover the binding pocket in some MD snapshots. Therefore we removed 9 residues of the MD snapshot Charmm_706ps (M1-H9) for further docking and binding free energy calculations.

#### Identification of Putative Binding Pockets

We performed interactive structural analysis of the minimized and the averaged MD trajectory mutant homo-dimer structures using a probe-mapping algorithm of Surflex-Protomol [71] (with CH4, C = O, and N-H groups as probes) to identify the zones capable of binding small-molecule ligands. In order to generate alternative conformations of the identified putative binging sites, we extracted 1500 snapshots at each 1ps timestep from the last 1500 ps of the MD trajectory of the mutant homo-dimer structure of SMS. Root Mean Square Deviations (RMSD) between the 1500 structures were calculated over all atoms of the putative binding sites (Table S3). We clustered the different conformations of the binding sites by applying the Hierarchical Ascendant Classification (HAC) on the obtained RMSD matrix using the aggregative method Ward as implemented in R (http://cran.r-project.org/) and a RMSD distance of at least 1.3Å. We took the centroid structure of the 8 obtained clusters in order to define a representative set of binding site conformations for further analysis.

We used the probe-mapping algorithm of Surflex-Protomol and the webserver DoGSiteScorer (http://dogsite.zbh.uni-hamburg.de/), to characterize the selected mutant dimer conformations [72]. DoGSiteScorer automatically detects druggable pockets by employing a support vector machine method and performing several pocket descriptor calculations. It returns a score of pocket druggability between 0 and 1 (0– non-druggable, 1– druggable). We applied DoGSiteScorer on the entire dimer structure to predict the druggable pockets and to compute pocket descriptors including volume, surface, lipophilicity, and druggability score.

#### Chemical Compound Collection

To provide valuable starting points for the virtual and in vitro screens, we prepared a diverse chemical compound collection. Four commercial libraries were assembled: Asinex Merged Libraries (436,012 compounds), ChemBridge Express Pick (324,909 compounds), ChemDiv Full Discovery Chemistry (1,183,665 compounds), and LifeChemicals Stocks (344,693 compounds). After removing the redundant molecules, we employed a drug-like filter using the FAF-Drugs2 web-service [73] previously developed in our lab in order to remove molecules with undesired physicochemical properties and reactive groups. It has been recently observed that the physicochemical properties of small molecules acting as protein-protein interaction (PPI) modulators [24], [74]–[76] differ from those defined by “Lipinski’s rule of 5” [77]. Such molecules are generally larger and more lipophilic. In order to increase the chance to find potent PPI small-molecule stabilizers while remaining drug-like, we decided to filter our compounds in the ranges: 100< MW (Molecular Weight) <700; 0< tPSA (topological polar surface area)<160; −4< logP<6; 0< number of HBD (hydrogen bond donors) <5; 0< number of HBA (hydrogen bond acceptors) <10; 0< Rotatable Bonds <15. The filtered collection contained 1,960,000 molecules that were clustered using the Cluster Molecule Protocol (Accelrys Pipeline Pilot v8.5) with the FCFP-4 fingerprint using a maximum distance of Tanimoto of 0.3 in the clusters. Tanimoto index of 0 means that there are no identical indices in either molecule and 1 means that both molecules are composed of identical sets of indices. The 3D structures of the remaining 273,226 molecules were generated using Corina program embedded in the Accelrys Pipeline Pilot v8.5. The procedure was launched keeping a maximum of 2 stereocenters and a maximum of 4 stereoisomers per compound without generating multiple ring conformations.

For chemical structural analysis of the identified bioactive compounds we used two clustering approaches. A first run was performed with the Cluster Molecule Protocol (Accelrys Pipeline Pilot v8.5) and the MDL keys. A Tanimoto similarity index of 0.6 was used to assess the similarity between all pair of compounds and 11 clusters were obtained. A second clustering procedure was carried out with Stardrop (http://www.optibrium.com/) in order to define a final chemical series. It creates chemical space projections based on a combination of chemical structure and properties.

#### Virtual Screening and Docking

Docking of the prepared 273,226 compounds from Asinex, ChemBridge, ChemDiv and LifeChemicals was performed into different protein binding pocket conformations using two software packages, Surflex [71] and AutoDock Vina [78]. Surflex creates a protomol of chemical probes to which potential ligands are aligned by incremental construction based on the molecular similarity. In this work, we generated Surflex protomol based on the selected residues in the binding pockets. The residue lists are provided in Table S4. In addition, the parameter “proto_thresh” was set to control the degree of burying (Table S5 of supporting information) and the parameter “proto_bloat” was set to indicate how far the protomol should be expanded (Table S5). During the docking process, the docking accuracy parameter (-pgeom) was used to start each docking run from 5 different initial poses to ensure good search coverage. We performed several post-processing runs to optimize the scoring parameters. The “polar” term was increased to 1.5; while the “penetration” term, was set to “−3.0” (default value). This term “−3.0” allows some protein-ligand atom overlaps, thereby permitting a slight “induced fit”.

AutoDock Vina employs a gradient-based conformational search approach and defines the search space by a grid box defined by the box center coordinates and its dimensions of x, y and z. We used grid resolution of 1 Å, number of binding modes of 10, and exhaustiveness of 8. The other parameters set used for running AutoDock Vina are provided in Table S6. The protein was prepared with the graphical user interface AutoDockTools (ADT) [79]. The grid enveloped the entire binding pocket surface of the targeted protein structures. The scoring of the generated docking poses and ranking of the ligands was based on the Vina empirical scoring function approximating the binding affinity in kcal/mol.

Additional docking experiments were executed with AutoDock4 [79] for further analysis of the binding modes of bioactive compounds, taking into account local receptor flexibility. We carried out docking in the binding zones Pa and Pb of the Charmm_706ps structure taken after the MD simulation of the complex Charmm_706ps - E941-0318 using a grid containing 44×80×100 grid points with a spacing of 0.375 Å. All torsions of the ligands and the side chains of the R17 (C chain) and H81 (D chain) were allowed to rotate. The Lamarckian genetic algorithm (LGA) was used to generate orientations/conformations of the compound. Thirty docking runs were performed, with an initial population of 150 random individuals and a maximum number of 25×10^6^ energy evaluations. The two mostly populated lowest energy positions were obtained for subpockets Pa and Pb.

#### Free Binding Energy-Based approach

The binding energy calculations were performed with CHARMM. The topology/parameter files for small molecules were created by the webserver Swissparam (http://swissparam.ch) [80]. For each complex containing the SMS dimer and a bound small molecule, we ran MD simulation with production step of 2 ns and then we calculated the average of the energies obtained for the last 20 complex structures taken from the last 20 ps. We used 2 protocols, noted as “rigid” (Eq.1) and “relaxed” (Eq.2) ones, to calculate the free binding energy ΔΔG_bind_ between the protein dimer and a small molecule:
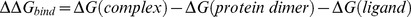
(1)where ΔΔG_bind_ is the free binding energy between the protein dimer and the ligand; ΔG(complex) is the potential energy of the complex; ΔG(protein dimer) is the potential energy of the protein dimer extracted from the complex; and ΔG(ligand) is the potential energy of the ligand extracted from the complex. The ΔΔG_bind-relaxed_ is computed using eq.2:
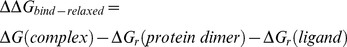
(2)where ΔΔG_bind-relaxed_ is the free binding energy between the protein dimer and the small molecule calculated by the relaxed approach; ΔG(complex) is the potential energy of the complex; ΔG_r_(protein dimer) is the potential energy of the protein dimer calculated from MD simulations performed on the protein dimer alone; and ΔG_r_(ligand) is the potential energy of the ligand calculated from MD simulations performed on the small molecule alone. The difference between (1) and (2) is that the energies in Eq.2 are calculated from 3 independent MD simulation runs for the complex, the protein dimer, and the ligand. The (1) and (2) were used to estimate the effect of small molecule binding on the stability of the dimer through the ligand binding energy taking into consideration that only the dimer interface zone was targeted by the ligands. The potential energy components include molecular mechanics energy, electrostatic interactions, and solvation energy. Entropic effects are implicitly taken into account in (2) because the MD simulation of the protein-ligand pair and the protein and ligands alone permits to account for entropy and dynamics [63].

## II. Experimental validation

### 

#### Production of G56S SMS mutant

A DNA fragment encoding human SMS was amplified by PCR and subcloned into the pQE-30 vector downstream of the polyhistidine coding region [81]. The resulting plasmids were used to transform XL1-Blue cells. Recombinant human SMS was purified by immobilized metal affinity chromatography using the TALON affinity resin (Clontech Laboratories, Palo Alto, CA, U.S.A.), in accordance with the manufacturer's instruction. The G56S mutated SMS was generated by PCR and subcloned into the pQE-30 vector. The entire coding sequence of the G56S SMS mutants was verified by DNA sequencing to ensure that no other mutations were introduced during PCR. The entire coding region of plasmids was verified by DNA sequencing carried out by the Macromolecular Core Facility, Hershey Medical Center.

#### *In vitro* assay of G56S SMS activity

The activity of G56S SMS mutants was measured in absence and in presence of small molecules. Details on the SMS activity measurements can be obtained from existing literature [82]. The small molecule candidates selected from the *in silico* analysis were dissolved in 5% BSA solution and the experiments were done with small molecules at 100 uM. The G56S mutant protein itself had little activity compared with WT (1.3% of WT activity) [51]. However, the addition of 5% BSA increased G56S mutant activity by 122.2% (a 2.22-fold increase in activity) [51]. Activity was measured by following the production of spermine from spermidine in 100 mM sodium phosphate buffer (pH 7.5) in the presence of 0.1 mM dcAdoMet as the propylamine donor. Reactions were run for 60 min and polyamines were extracted in 10% trichloroacetic acid. The extracts were directly injected onto the o-phthalaldehyde postcolumn ion-exchange HPLC system [83]. The activity measurements were performed two times and no differences were found.

## Supporting Information

Figure S1 **RMSD for backbone atoms between the MD trajectory and the minimized average structure (Red: WT; Black: Mutant G56S).** The production time was set to 2000 steps and each timestep is 1ps.
(TIFF)

Figure S2 **RMSD for backbone atoms between the 1500 conformations used for the HAC analysis and Charmm_706ps.**
(TIFF)

Figure S3 **Experimental activities (in %, the horizontal axis) and computed ΔΔG_bind_ (in red rectangles) and ΔΔG_bind-relaxed_ (in blue diamonds) energies (in kcal/mol, the vertical axis).**
(TIFF)

Figure S4 **Lowest docking energy conformations of Cluster IV bioactive compounds docked with AutoDock into the area Pa of Charmm_706ps taken after the MD simulation of the complex Charmm_706ps - E941-0318.** The C chain in shown in green, the D chain is shown in cyan. (A) docked E941-0318 and G796-1817 superposed into the Connolly surface of the dimer G56S SMS; (B) docked E941-0318; (C) docked G796-1817.
(TIFF)

Figure S5 **RMSF during MD simulations of mutant G56S (black), mutant G56S bound to the compound 9129729 (red) and mutant G56S bound to the compound E941-0318 (green).** Note that the residue numbers in D chain, which includes 381 amino acids as C chain, were counted from No. 382 to No. 762. The mutation site G56S in both C chain and D chain is pointed to by the blue arrow.
(TIFF)

Table S1 **Druggable pockets (P) close to the mutation site G56S identified by DoGSiteScorer for the 8 centroid structures obtained after HAC.**
(DOCX)

Table S2 **The list of deprotonated His based on the pKa calculation and residue analysis of 3D structure.**
(DOCX)

Table S3 **The selected residues of the putative binding sites.**
(DOCX)

Table S4 **Residue list for generating the protomol for docking with Surflex.**
(DOCX)

Table S5 **Parameters to control the degree of burying (proto_thresh) and extention (proto_bloat) of the protomol.**
(DOCX)

Table S6 **The coordinates of the grid box center and the dimension of the grid box used for docking with AutoDock Vina.**
(DOCX)
