# Supplementary figures and images for: Rational Design of Small-Molecule Stabilizers of Spermine Synthase Dimer by Virtual Screening and Free Energy-Based Approach
Source: PLoS One. 2014 Oct 23;9(10):e110884. doi: 10.1371/journal.pone.0110884 (PMC4207787; doi:10.1371/journal.pone.0110884)

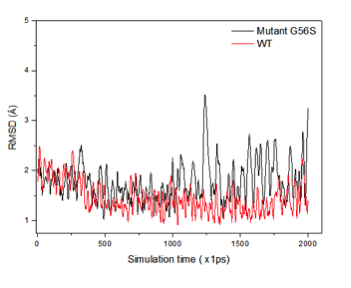

Supplement: Figure S1 — RMSD for backbone atoms between the MD trajectory and the minimized average structure (Red: WT; Black: Mutant G56S). The production time was set to 2000 steps and each timestep is 1ps. (TIFF) [file pone.0110884.s001.tiff]

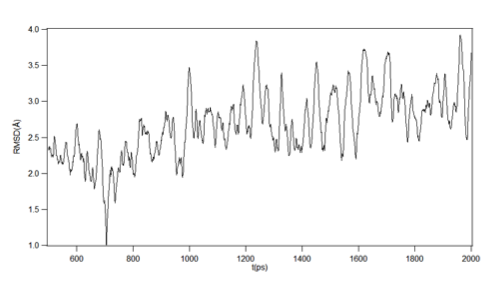

Supplement: Figure S2 — RMSD for backbone atoms between the 1500 conformations used for the HAC analysis and Charmm_706ps. (TIFF) [file pone.0110884.s002.tiff]

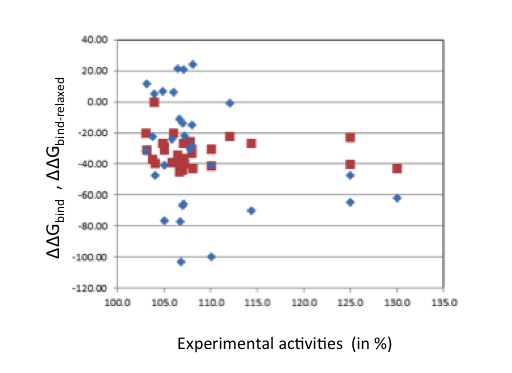

Supplement: Figure S3 — Experimental activities (in %, the horizontal axis) and computed ΔΔGbind (in red rectangles) and ΔΔGbind-relaxed (in blue diamonds) energies (in kcal/mol, the vertical axis). (TIFF) [file pone.0110884.s003.tiff]

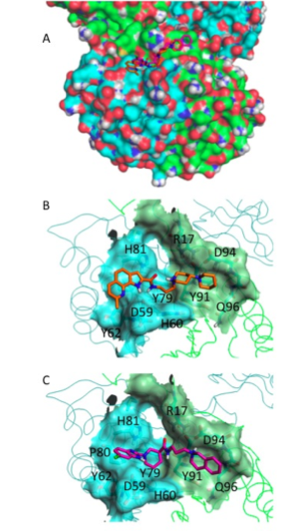

Supplement: Figure S4 — Lowest docking energy conformations of Cluster IV bioactive compounds docked with AutoDock into the area Pa of Charmm_706ps taken after the MD simulation of the complex Charmm_706ps - E941-0318. The C chain in shown in green, the D chain is shown in cyan. (A) docked E941-0318 and G796-1817 superposed into the Connolly surface of the dimer G56S SMS; (B) docked E941-0318; (C) docked G796-1817. (TIFF) [file pone.0110884.s004.tiff]

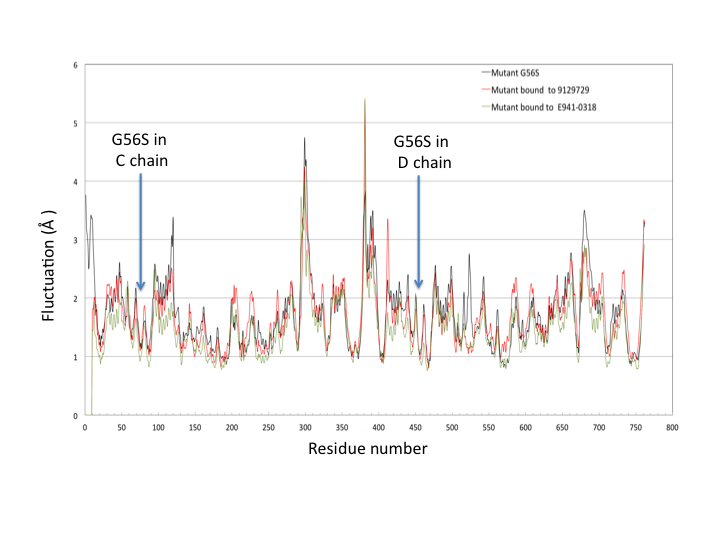

Supplement: Figure S5 — RMSF during MD simulations of mutant G56S (black), mutant G56S bound to the compound 9129729 (red) and mutant G56S bound to the compound E941-0318 (green). Note that the residue numbers in D chain, which includes 381 amino acids as C chain, were counted from No. 382 to No. 762. The mutation site G56S in both C chain and D chain is pointed to by the blue arrow. (TIFF) [file pone.0110884.s005.tiff]
