# Supplementary material for: Rational Design of Small-Molecule Stabilizers of Spermine Synthase Dimer by Virtual Screening and Free Energy-Based Approach
Source: PLoS One. 2014 Oct 23;9(10):e110884. doi: 10.1371/journal.pone.0110884 (PMC4207787; doi:10.1371/journal.pone.0110884)
Supplement: Table S1 — Druggable pockets (P) close to the mutation site G56S identified by DoGSiteScorer for the 8 centroid structures obtained after HAC. (DOCX) [file pone.0110884.s006.docx]

**Table S1.** Druggable pockets (P) close to the mutation site G56S identified by DoGSiteScorer for the 8 centroid structures obtained after HAC

| **Pocket** | **Volume [Å³]** | **Surface**  **[Å²]** | **Solvent accessible lipophilic surface [Å²]** | **Drugability Score** |
| --- | --- | --- | --- | --- |
| Charmm_630ps |  |  |  |  |
| P3 | 443.47 | 682.25 | 378.20 | 0.66 |
| P22 | 125.86 | 269.05 | 165.48 | 0.19 |
|  |  |  |  |  |
| Charmm_661ps |  |  |  |  |
| P27 | 105.37 | 165.38 | 117.19 | 0.19 |
|  |  |  |  |  |
| Charmm_668ps |  |  |  |  |
| P4 | 436.80 | 627.49 | 315.60 | 0.69 |
| P5 | 396.22 | 712.17 | 496.64 | 0.61 |
| P26 | 103.26 | 126.49 | 96.70 | 0.27 |
|  |  |  |  |  |
| Charmm_706ps |  |  |  |  |
| P0 | 1490.59 | 1875.12 | 1296.21 | 0.81 |
| P4 | 536.92 | 458.24 | 284.69 | 0.84 |
|  |  |  |  |  |
| Charmm_790ps |  |  |  |  |
| P2 | 808.66 | 861.46 | 592.88 | 0.84 |
| P13 | 188.67 | 350.19 | 181.55 | 0.32 |
| P26 | 110.05 | 117.60 | 88.65 | 0.28 |
|  |  |  |  |  |
| Charmm_1139ps |  |  |  |  |
| P4 | 506.62 | 415.71 | 300.41 | 0.74 |
| P8 | 291.49 | 510.71 | 322.64 | 0.56 |
| P17 | 139.04 | 237.33 | 131.89 | 0.33 |
|  |  |  |  |  |
| Charmm_1165ps |  |  |  |  |
| P5 | 412.23 | 610.88 | 371.49 | 0.66 |
| P7 | 238.46 | 417.92 | 186.31 | 0.41 |
|  |  |  |  |  |
| Charmm_1353ps |  |  |  |  |
| P2 | 808.66 | 861.46 | 592.88 | 0.84 |
| P13 | 188.67 | 350.19 | 181.55 | 0.32 |
| P26 | 110.05 | 117.60 | 88.65 | 0.28 |
