# Supplementary material for: Rational Design of Small-Molecule Stabilizers of Spermine Synthase Dimer by Virtual Screening and Free Energy-Based Approach
Source: PLoS One. 2014 Oct 23;9(10):e110884. doi: 10.1371/journal.pone.0110884 (PMC4207787; doi:10.1371/journal.pone.0110884)
Supplement: Table S2 — The list of deprotonated His based on the pKa calculation and residue analysis of 3D structure. (DOCX) [file pone.0110884.s007.docx]

**Table S2.** The list of deprotonated His based on the pKa calculation and residue analysis of 3D structure

| Residue Number | pKa Value | Protonation |
| --- | --- | --- |
| HIS5 C chain | 4.43 | ε protonation |
| HIS6 C chain | 6.24 | δ protonation |
| HIS8 C chain | 5.85 | ε protonation |
| HIS9 C chain | 6.49 | δ protonation |
| HIS21 C chain | 1.05 | ε protonation |
| HIS55 C chain | 4.26 | δ protonation |
| HIS5 D chain | 0.0 | ε protonation |
| HIS6 D chain | 5.57 | δ protonation |
| HIS8 D chain | 5.08 | ε protonation |
| HIS9 D chain | 0.72 | δ protonation |
| HIS21 D chain | 2.44 | ε protonation |
| HIS55 D chain | 3.72 | δ protonation |
