# Supplementary material for: Rational Design of Small-Molecule Stabilizers of Spermine Synthase Dimer by Virtual Screening and Free Energy-Based Approach
Source: PLoS One. 2014 Oct 23;9(10):e110884. doi: 10.1371/journal.pone.0110884 (PMC4207787; doi:10.1371/journal.pone.0110884)
Supplement: Table S3 — The selected residues of the putative binding sites. (DOCX) [file pone.0110884.s008.docx]

**Table S3.** The selected residues of the putative binding sites

| 3C6K (rebuilt by profix) | | 3C6K (Original PDB file) | |
| --- | --- | --- | --- |
| C Chain | D Chain | C Chain | D Chain |
| S11 | M27 | N/A | M14 |
| G13 | A32 | N/A | N/A |
| L14 | D33 | N/A | D20 |
| P16 | G34 | N/A | G21 |
| R17 | E35 | N/A | E22 |
| G18 | Q58 | G5 | Q45 |
| G71 | D59 | G58 | D46 |
| Y91 | H60 | Y78 | H47 |
| D92 | G61 | D79 | G48 |
| G93 | Y62 | N/A | Y49 |
|  | R77 |  | R64 |
|  | I78 |  | I65 |
|  | Y79 |  | Y66 |
|  | P80 |  | P67 |
|  | H81 |  | H68 |

N/A: Residues were missed in the original PDB file and were rebuilt with profix;

The corresponding residue number of the mutation site G56 reported in the literature and is G58 in 3C6K (original PDB file) and G71 in 3C6K (rebuilt structure) respectively.
