# Supplementary material for: Rational Design of Small-Molecule Stabilizers of Spermine Synthase Dimer by Virtual Screening and Free Energy-Based Approach
Source: PLoS One. 2014 Oct 23;9(10):e110884. doi: 10.1371/journal.pone.0110884 (PMC4207787; doi:10.1371/journal.pone.0110884)
Supplement: Table S4 — Residue list for generating the protomol for docking with Surflex. (DOCX) [file pone.0110884.s009.docx]

**Table S4**. Residue list for generating the protomol for docking with Surflex

| **Charmm_mini** |  | **Charmm_ave** |  | **Charmm_706ps** |  |
| --- | --- | --- | --- | --- | --- |
| **C chain** | **D chain** | **C chain** | **D chain** | **C chain** | **D chain** |
| S11 | M27 | H5 | A32 | L14 | M27 |
| S12 | L28 | S11 | D33 | P16 | L28 |
| V15 | G29 | S12 | G34 | R17 | G29 |
| P16 | A30 | L14 | E35 | G18 | A30 |
| R17 | A32 | P16 | I37 | S19 | K31 |
| N70 | D33 | R17 | Q58 | S71 | A32 |
| S71 | G34 | G18 | D59 | Y91 | D33 |
| Y91 | E35 | S19 | H60 | D92 | G34 |
| D92 | I37 | S71 | G61 | G93 | E35 |
| A95 | D59 | Y91 | Y62 |  | I37 |
| Q96 | H60 | D92 | R77 |  | Q58 |
|  | G61 | G93 | I78 |  | D59 |
|  | Y62 | A95 | Y79 |  | H60 |
|  | I78 |  | P80 |  | G61 |
|  | Y79 |  | H81 |  | Y62 |
|  | P80 |  | G82 |  | R77 |
|  | H81 |  |  |  | I78 |
|  | G82 |  |  |  | Y79 |
|  |  |  |  |  | P80 |
|  |  |  |  |  | H81 |
|  |  |  |  |  | G82 |

Charmm_mini: Minimized structure;

Charmm_ave: Average MD minimized structure;

Charmm_706ps: The snap shot at 706ps.
