# Supplementary material for: Rational Design of Small-Molecule Stabilizers of Spermine Synthase Dimer by Virtual Screening and Free Energy-Based Approach
Source: PLoS One. 2014 Oct 23;9(10):e110884. doi: 10.1371/journal.pone.0110884 (PMC4207787; doi:10.1371/journal.pone.0110884)
Supplement: Table S5 — Parameters to control the degree of burying (proto_thresh) and extention (proto_bloat) of the protomol. (DOCX) [file pone.0110884.s010.docx]

**Table S5.** Parameters to control the degree of burying (proto_thresh) and extention (proto_bloat) of the protomol

|  | Charmm_mini | Charmm_ave | Charmm_706ps |
| --- | --- | --- | --- |
| proto_thresh | 0.6 | 0.5 | 0.6 |
| proto_bloat | 5 | 5 | 4 |

Charmm_mini: Minimized structure;

Charmm_ave: Average MD minimized structure;

Charmm_706ps: The snap shot at 706ps;
