# Supplementary material for: Rational Design of Small-Molecule Stabilizers of Spermine Synthase Dimer by Virtual Screening and Free Energy-Based Approach
Source: PLoS One. 2014 Oct 23;9(10):e110884. doi: 10.1371/journal.pone.0110884 (PMC4207787; doi:10.1371/journal.pone.0110884)
Supplement: Table S6 — The coordinates of the grid box center and the dimension of the grid box used for docking with AutoDock Vina. (DOCX) [file pone.0110884.s011.docx]

**Table S6**. The coordinates of the grid box center and the dimension of the grid box used for docking with AutoDock Vina

| Structures |  | Charmm_mini | Charmm_ave | Charmm_706ps |
| --- | --- | --- | --- | --- |
| Center  Coordinates | x | 9.546 | 19.242 | 17.738 |
|  | y | 29.052 | 39.173 | 37.35 |
|  | z | -90.227 | -94.053 | -95.176 |
| Dimension | x | 26 | 14 | 12 |
|  | y | 30 | 28 | 25 |
|  | z | 29 | 20 | 20 |
